# Temperature regulation of a nonlinear CSTR using a global-guided optimization-based PID framework

**DOI:** 10.1038/s41598-026-57648-2

**Published:** 2026-06-14

**Authors:** Cebrail Turkeri, Serdar Ekinci, Davut Izci, Dacheng Li, Emine Ayaz, Vedat Tumen, Erdal Akin

**Affiliations:** 1 Department of Computer Engineering, Batman University, Batman, 72100 Türkiye Turkey; 2 Department of Computer Engineering, Bitlis Eren University, Bitlis, 13100 Türkiye Turkey; 3 Department of Electrical and Electronics Engineering, Uludag University, Bursa, 16059 Türkiye Turkey; 4 Birmingham Centre for Energy Storage, School of Chemical Engineering, University of Birmingham, Edgbaston, B15 2TT UK; 5 Department of Computer Science and Media Technology, Malmö University, Malmö, 205 06, Sweden; 6 Sustainable Digitalisation Research Centre, Malmö University, Malmö, 205 06 Sweden; 7 Biofilms Research Center for Biointerfaces (BRCB), Malmö University, Malmö, Sweden

**Keywords:** Continuous stirred tank reactor, Nonlinear systems, PID controller, Optimization-based tuning, Performance evaluation, Energy science and technology, Engineering, Mathematics and computing

## Abstract

Accurate temperature regulation in nonlinear continuous stirred tank reactors (CSTRs) remains a challenging task due to strong nonlinearities and operating-point sensitivity. Although numerous proportional-integral-derivative (PID) tuning approaches have been proposed, most existing studies primarily focus on nominal operating conditions, often resulting in degraded performance under varying process dynamics. To address this limitation, this study proposes an optimization-based PID with filter (PIDf) tuning framework that enhances consistency and reliability across different operating scenarios. A global-guided search mechanism is incorporated into the optimization process to improve convergence stability and solution quality without increasing computational complexity. The proposed framework is evaluated on a nonlinear jacketed CSTR system under setpoint variations and multiple operating conditions. Its performance is benchmarked against recent metaheuristic optimization methods and classical tuning strategies using time-domain specifications and error-based performance indices. The results indicate that the proposed approach achieves faster settling behavior, reduced overshoot, and improved consistency, while maintaining stable performance across repeated runs. Overall, the performance of the proposed approach is quantitatively assessed using standard time-domain and error-based metrics, providing a systematic evaluation of control quality. These findings highlight the applicability of the proposed framework for temperature regulation in nonlinear chemical processes.

## Introduction

Continuous stirred tank reactors (CSTRs) constitute one of the most fundamental and widely employed process units in chemical and related industries due to their simplicity, continuous operation, and ease of integration into large-scale production systems. In many industrial applications, CSTRs are used to carry out highly exothermic reactions, where maintaining the reactor temperature within a narrow operating range is critical for ensuring product quality, operational safety, and energy efficiency. However, the intrinsic coupling between reaction kinetics and heat-transfer mechanisms gives rise to strongly nonlinear dynamics, making temperature regulation a challenging control problem, particularly in the presence of disturbances and operating-point variations^1,2^. Small deviations in reactor temperature may lead to significant changes in reaction rates, which can result in undesirable transient behavior or, in extreme cases, thermal runaway^3^. For this reason, accurate and robust temperature regulation has long been regarded as a central requirement in CSTR operation, motivating sustained research efforts on control-oriented modelling and regulation strategies for nonlinear reactor systems.

A broad spectrum of advanced control methodologies has been investigated for CSTR temperature regulation^4,5^. For instance, model predictive control (MPC) has attracted considerable attention due to its ability to explicitly incorporate process constraints and future-state predictions, yielding improved tracking performance in nonlinear reactor systems^6,7^. Recently, Wu et al.^8^ proposed a fast predictive functional control strategy based on auxiliary variable optimization for complex chemical processes with large time delays and high inertia, demonstrating the potential of enhanced predictive control structures to reduce online computational burden while improving tracking performance. In parallel, adaptive control frameworks have been developed to cope with time-varying dynamics and parametric uncertainties by updating controller parameters online^9^. Robust control-oriented strategies have also been explored for CSTR-related processes to strengthen disturbance attenuation and reduce sensitivity to modelling errors. In particular, sliding mode control (SMC) and its PID-based variants have been widely employed in nonlinear process control due to their robustness properties and strong disturbance-rejection capability^10,11^. Likewise, H∞-based robust designs have been reported for reactor-type applications to address uncertainty and performance requirements within a formal synthesis framework^12^. Despite their advantages, such methods may entail nontrivial design effort and implementation considerations, for example, chattering mitigation in SMC or conservatism in robust synthesis, especially in strongly nonlinear thermal systems.

More recently, intelligent and data-driven control paradigms have gained prominence for exothermic reactor regulation, motivated by the need to cope with pronounced nonlinearities and partially unknown dynamics. Fuzzy logic controllers and fuzzy-PID structures have been investigated to incorporate heuristic operating knowledge into the control law^13,14^. Neural-network-based strategies, including neuro-PID and learning-based tracking controllers, have also been adopted to enhance approximation and tracking performance in CSTR settings^15,16^. In practice, however, the industrial deployment of these advanced strategies can be constrained by computational burden, implementation cost, data dependency, and limited transparency^17^. Consequently, PID-based controller architectures, including their widely adopted structural extensions, remain the dominant choice in industrial process applications, including CSTR operation^18,19^. This motivates continued interest in enhancing PID-type designs through tuning strategies that preserve practical simplicity while improving robustness and performance^20,21^.

Nevertheless, achieving reliable and consistent performance with classical PID controllers in CSTR applications remains challenging. The highly nonlinear behavior of exothermic reactors, together with sensitivity to operating-point variations, model uncertainties, and external disturbances, can degrade control performance when fixed-gain PID parameters are employed^22^. Conventional tuning rules, such as Ziegler-Nichols^23,24^ and other classical approaches, while effective for linear or weakly nonlinear systems, may lead to excessive overshoot, prolonged settling times, or reduced robustness when applied to strongly nonlinear thermal processes such as CSTRs^25^. These limitations have motivated sustained efforts toward optimization-based and adaptive tuning methodologies capable of handling nonlinear dynamics and varying operating conditions.

To overcome the limitations of fixed-parameter tuning in nonlinear reactor systems, metaheuristic optimization algorithms and improved variants that incorporate enhanced search behaviors, have gained increasing attention due to their derivative-free nature, flexibility, and ability to handle nonconvex and multimodal performance landscapes^26–32^. These characteristics make metaheuristic approaches particularly attractive for strongly nonlinear thermal processes such as CSTRs. In line with this trend, several metaheuristic algorithms proposed in 2025 have demonstrated promising performance in complex engineering optimization tasks, including controller parameter tuning. Among these, the artificial lemming algorithm (ALA)^33^, tornado optimizer with coriolis force (TOC)^34^, mirage search optimization (MSO)^35^, and phototropic growth algorithm (PGA)^36^ have attracted attention due to their bio-inspired search mechanisms and competitive convergence behavior. However, their convergence characteristics and exploitation capability may vary with problem structure, particularly in strongly nonlinear and disturbance-sensitive control applications such as CSTR temperature regulation.

A common challenge in population-based metaheuristics is balancing global exploration and local exploitation^37^. While stochastic interactions and local search behaviors promote diversity, the lack of explicit global guidance can lead to slow convergence or stagnation around suboptimal regions in highly nonlinear control landscapes. To address this issue, global-best (gbest) learning concepts, widely used in swarm-based optimization frameworks, have been reported to enhance exploitation capability and convergence stability by guiding individuals toward collectively identified high-quality solutions^38–40^.

Despite the growing use of metaheuristic-based PID tuning approaches in nonlinear process control, most existing studies primarily focus on performance improvement under nominal operating conditions. However, nonlinear CSTR systems are inherently sensitive to operating-point variations, disturbances, and parameter uncertainties, which may significantly degrade controller performance. In addition, the consistency and robustness of optimization-based tuning strategies across different operating scenarios remain insufficiently addressed in the literature. Therefore, there is a clear need for a robust and reliable tuning framework capable of maintaining stable and consistent performance under varying process conditions.

Motivated by these observations, this study proposes an enhanced gbest-ALA for the optimal tuning of a PID controller with filter (PIDf) applied to nonlinear CSTR temperature regulation. To the authors’ best knowledge, integrating a global-best guidance mechanism into the artificial lemming algorithm and employing it for PIDf-based CSTR control has not been previously reported, which will greatly enhance the robustness and reliability of the CSTR operation. The proposed approach is evaluated on a nonlinear jacketed CSTR and benchmarked against recently introduced 2025 metaheuristics as well as classical PID tuning methods.

The main contributions of this study can be summarized as follows. A robust PIDf tuning framework is developed for nonlinear CSTR temperature control under operating-point variations. A global-guided search mechanism is incorporated to enhance convergence stability and improve solution consistency. The proposed approach is systematically evaluated under different operating conditions to demonstrate robustness and reliability. In addition, a comprehensive comparative analysis is conducted against recent metaheuristic and classical tuning methods using time-domain and statistical performance criteria.

The remainder of this paper is organized as follows. Section 2 presents an overview of the original artificial lemming algorithm. Section 3 introduces the proposed gbest-ALA and describes the integration of the global-best guidance mechanism. Section 4 provides the nonlinear CSTR model, including the reaction kinetics, dynamic balance equations, and steady-state operating point. Section 5 describes the PIDf controller structure used for temperature regulation. Section 6 presents the simulation results, comparative analyses, statistical evaluations, and discussion of the obtained findings. Finally, Sect. 7 concludes the paper and outlines possible future research directions.

## Overview of artificial lemming algorithm (ALA)

The artificial lemming algorithm (ALA)^33^ is a bio-inspired meta-heuristic optimization algorithm developed to solve complex optimization problems. The algorithm aims to establish a dynamic balance between exploration and exploitation in the search space by mimicking the survival and resource-seeking behaviors of lemmings. ALA obtains a population matrix by randomly positioning * M* number of search agents in the optimization space before starting the optimization process. Equation (1) gives the set of all initial candidate solutions.

1 $$\:\overrightarrow{X}=\left[\begin{array}{ccc}{x}_{\mathrm{1,1}}&\:\cdots\:&\:{x}_{1,D}\\\:\vdots&\:\ddots\:&\:\vdots\\\:{x}_{M,1}&\:\cdots\:&\:{x}_{M,D}\end{array}\right]$$

The best position in each iteration is considered to be the most suitable solution obtained up to that point or the closest to the most suitable solution. For each dimension, the decision variables $\:{x}_{p,q}$ are calculated using Eq. (2).

2 $$\:{x}_{p,q}=\:{L}_{q\:}+\:r\cdot\:\:({U}_{q}-\:{L}_{q}),\:p=1,\:2,...,M,\:q=1,\:2,\:.\:.\:.\:,\:D$$

Here, * r* is a random number in the range [0,1], $\:{L}_{q}$ is the lower bound of the $\:{q}^{th}$ dimension, $\:{U}_{q}$ is the upper bound of the $\:{q}^{th}$ dimension, * M* is the population size, and * D* is the number of dimensions. It uses an energy reduction mechanism (* Eng*) that determines when search agents will explore and when they will engage in local exploitation. This mechanism models the natural responses of lemmings to resource scarcity and predator threats. Search agents perform exploration behaviors, including long-distance migration or hole digging, when they have sufficient energy (* Eng>1*). Otherwise ($\:Eng\le\:1$), they perform exploitation behaviors, including predator avoidance or food search. Additionally, the probability of * Eng>1* or $\:Eng\le\:1$ is used to support the algorithm’s transition from exploration to exploitation. $\:Eng\left(t\right)$ is calculated using Eq. (3).

3 $$\:Eng\left(t\right)=4\cdot\:arctan\left[1-\frac{1}{{T}_{max}}\right]\cdot\:ln\left(\frac{1}{r}\right)$$

Position updates in ALA are based on the four fundamental behaviors of lemmings. $\:{\overrightarrow{x}}_{p}^{(t+1)}$ indicates the agent’s new position in the next iteration. The exploration phase (* Eng>1*) aims to find new regions by scanning the entire search space. This behavior simulates the lemmings’ movement in a random direction relative to the current best solution ($\:{\overrightarrow{x}}_{best}$) and is enhanced by Brownian motion (* G*). The agent’s position in iteration (* t+1*) is updated using Eq. (4).

4 $$\:{\overrightarrow{x}}_{p}^{(t+1)}={\overrightarrow{x}}_{best}^{\left(t\right)}+\mathrm{s}\cdot\:\:\mathrm{G}\cdot\:\left(\overrightarrow{{\uprho\:}}\cdot\:({\overrightarrow{x}}_{best}^{\left(t\right)}-{\overrightarrow{x}}_{p}^{\left(t\right)})+(1-\overrightarrow{{\uprho\:}})\cdot\:({\overrightarrow{x}}_{p}^{\left(t\right)}-{\overrightarrow{x}}_{rnd}^{\left(t\right)})\right)$$

Here, $\:{\overrightarrow{x}}_{p}^{\left(t\right)}$ is the position vector of the $\:{p}^{th}$ agent, * t* is the current iteration, $\:{\overrightarrow{x}}_{best}^{\left(t\right)}$ is the position of the current optimal individual in the population, and $\:{\overrightarrow{x}}_{rnd}^{\left(t\right)}$ is the position of another randomly selected individual from the population, * s* is the migration/influence coefficient, * G* is the influence vector per dimension, *ρ* ∈ [0,1]: the direction/decision weight factor *ρ* determines the mix between convergence (towards the best) and escape.

Digging holes serves the purpose of avoiding predators and searching for better food. This action allows the agent to take a random step using its current position $\:{\overrightarrow{x}}_{p}^{\left(t\right)}$ and $\:{\overrightarrow{x}}_{best}^{\left(t\right)}$, the best known position. The new position is updated using Eq. (5).

5 $$\:{\overrightarrow{x}}_{p}^{(t+1)}={\overrightarrow{x}}_{p}^{\left(t\right)}+s\cdot\:\mathcal{l}\left(\mathrm{t}\right)\cdot\:({\overrightarrow{x}}_{best}^{\left(t\right)}-{\overrightarrow{x}}_{b}^{\left(t\right)})$$

Here, $\:\mathcal{l}\left(\mathrm{t}\right)$ is the random effect coefficient dependent on the current iteration (Eq. (6)), and $\:{\overrightarrow{x}}_{b}^{\left(t\right)}$ is the position of a randomly selected agent from the population ($\:{b}^{th}$ index).

6 $$\:\mathcal{l}\left(t\right)\:=r\cdot\:\:\left(1+sin\left(\frac{t}{2}\right)\right),\:r\:\in\:\:[0,\:1]$$

The exploitation phase focuses on locally improving the solution in promising areas found for foraging and avoiding predators. The new position $\:{\overrightarrow{x}}_{p}^{(t+1)}$ during foraging behavior is defined by a spiral movement relative to the current best solution $\:{\overrightarrow{x}}_{best}^{\left(t\right)}$. Foraging behavior is given by Eq. (7).

7 $$\:{\overrightarrow{x}}_{p}^{(t+1)}={\overrightarrow{x}}_{best}^{\left(t\right)}+s\cdot\:spiral\cdot\:r\cdot\:{\overrightarrow{x}}_{p}^{\left(t\right)}$$

Here, the spiral shape represents the random search during the spiral food search. The Lévy flight ($\:Levy\left(D\right)$) strategy is used for this phase. In the predator avoidance behavior, agents move around the current best solution using a Lévy random walk to optimize local exploitation. The position update is performed as in Eq. (8).

8 $$\:{\overrightarrow{x}}_{p}^{(t+1)}={\overrightarrow{x}}_{best}^{\left(t\right)}+s\cdot\:C\cdot\:Levy\left(D\right)\cdot\:({\overrightarrow{x}}_{best}^{\left(t\right)}-{\overrightarrow{x}}_{p}^{\left(t\right)})$$

$\:Levy\left(D\right)$ is a Lévy flight random number vector. * C* is an escape coefficient that models the escape ability of lemmings and decreases as the number of iterations increases, calculated as in Eq. (9). $\:{T}_{max}$ represents the maximum number of iterations.

9 $$\:C=2\cdot\:\left(1-\frac{t}{{T}_{max}}\right)$$

## Proposed gbest operator based artificial lemming algorithm (gbest-ALA)

### Motivation for integrating the gbest operator

The ALA provides an effective exploration-exploitation mechanism by simulating different behavioral patterns of lemmings, such as migration, digging, foraging, and predator avoidance. Although these operators enable diverse search trajectories, the original ALA may still experience slow convergence or premature stagnation when the promising regions of the search space are not emphasized sufficiently. To address this limitation, a global attraction mechanism inspired by the particle swarm optimization (PSO) framework is incorporated into the algorithm.

In PSO, the gbest operator directs each particle toward the best solution discovered so far, thereby reinforcing exploitation without discarding exploration. This mechanism has recently been adopted as an enhancement strategy in modern meta-heuristics. Notably, Abualigah et al.^38^ demonstrated that embedding a gbest-driven refinement step into the reptile search algorithm significantly improved solution quality and stability across a wide range of engineering problems. Their findings indicate that the gbest concept serves as a lightweight yet powerful operator that can be integrated into population-based optimizers without altering their internal behavioral logic.

Motivated by this evidence, ALA is augmented with a gbest operator to strengthen its local search capability while preserving the diversity introduced by its natural behaviors. In the proposed structure, each lemming follows the classical ALA movement rules, and subsequently receives a small corrective displacement toward the best-performing agent. This hybridization results in improved convergence reliability, accelerated fitness reduction, and a more balanced search progression.

### Mathematical formulation of the gbest-enhanced update

Let $\:{\stackrel{\sim}{x}}_{i}^{t+1}$ denote the preliminary position of the * i*-th search agent after completing one of the classical ALA behavioral updates at iteration t. In the proposed gbest-ALA, this preliminary position is further refined using a gbest-guided update mechanism inspired by the PSO-based formulation reported in Ref^38^. In this mechanism, the movement of each search agent is influenced by both its own best previously visited position and the global-best solution discovered by the whole population. Accordingly, the gbest-enhanced refinement is formulated as follows:

10 $$\:\begin{array}{c}{v}_{i}^{t+1}=\omega\:{v}_{i}^{t}+{c}_{1}{r}_{1}\left({pbest}_{i}^{t}-{\stackrel{\sim}{x}}_{i}^{t+1}\right)+{c}_{2}{r}_{2}\left({xbest}^{i}-{\stackrel{\sim}{x}}_{i}^{t+1}\right)\\\:{x}_{i}^{t+1}={\stackrel{\sim}{x}}_{i}^{t+1}+{v}_{i}^{t+1}\end{array}$$

where $\:{v}_{i}^{t}$ and $\:{v}_{i}^{t+1}$ denote the previous and updated velocity-like displacement vectors of the * i*-th search agent, respectively. The parameter $\:\omega\:$ represents the inertia weight, while $\:{c}_{1}$ and $\:{c}_{2}$ are acceleration coefficients that control the effects of individual learning and global learning, respectively. The terms $\:{r}_{1}$ and $\:{r}_{2}$ are random numbers uniformly distributed in the interval [0,1]. Moreover, $\:{pbest}_{i}^{t}$ denotes the best position previously visited by the * i*-th search agent, whereas $\:{xbest}^{i}$ represents the global-best solution found by the whole population up to iteration * t*. In this study, the ALA-generated preliminary position $\:{\stackrel{\sim}{x}}_{i}^{t+1}$ is used as the base candidate solution to which the gbest-guided refinement is applied.

This formulation enables the proposed gbest-ALA to preserve the original behavioral search dynamics of ALA while introducing an additional memory-guided exploitation mechanism. The personal-best component allows each search agent to benefit from its own successful search history, whereas the global-best component guides the population toward the most promising region identified so far. As a result, the proposed update improves local refinement and convergence reliability without discarding the stochastic exploration capability provided by the original ALA operators.

### Overall workflow of the proposed gbest-ALA

**Fig. 1:**
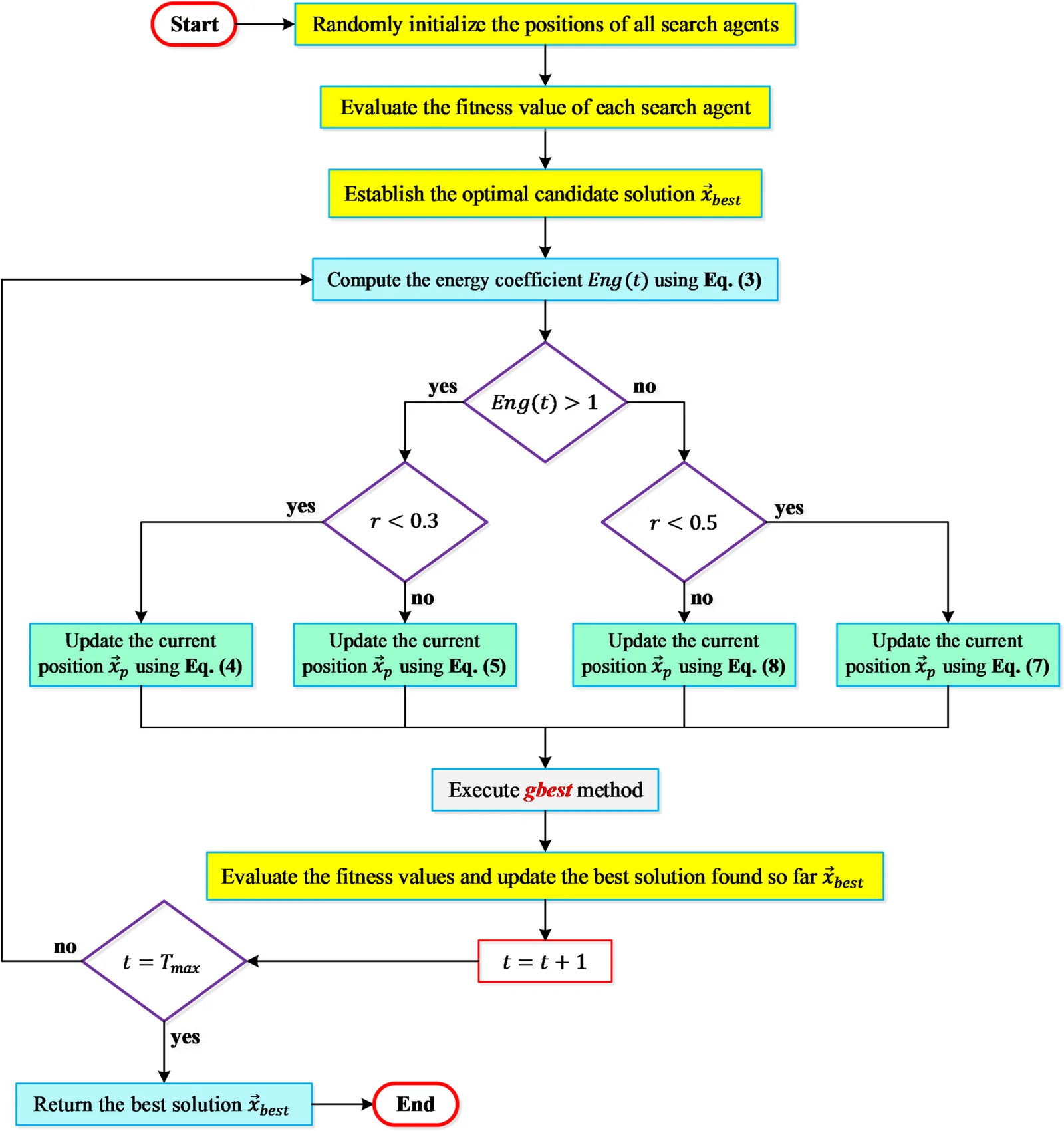
Flowchart of gbest-ALA.

The overall computational structure of gbest-ALA is illustrated in Fig. 1. The algorithm begins by initializing the positions of all search agents and evaluating their corresponding objective function values. At each iteration, the energy coefficient $\:Eng\left(t\right)$, computed using Eq. (3), determines the behavioral mode of each search agent: migration (Eq. (4)), digging (Eq. (5)), foraging (Eq. (7)), or predator-avoidance exploitation (Eq. (8)). These mechanisms constitute the core dynamic operations of ALA and generate a preliminary candidate position $\:{\stackrel{\sim}{x}}_{i}^{t+1}$ for each search agent.

After the ALA-based behavioral update, the proposed gbest-guided refinement step is applied using Eq. (10). In this stage, a velocity-like displacement vector is calculated by considering the previous displacement of the agent, its personal-best position $\:{pbest}_{i}^{t}$, and the global-best solution $\:{xbest}^{i}$. The final updated position $\:{x}_{i}^{t+1}$ is then obtained by adding this displacement to the ALA-generated preliminary position $\:{\stackrel{\sim}{x}}_{i}^{t+1}$. By doing so, the algorithm preserves ALA’s intrinsic exploratory diversity while introducing a directed exploitation mechanism that encourages search agents to move toward historically promising regions of the search landscape.

Following the gbest-guided refinement, boundary control is applied when necessary, and the fitness values of the refined candidate solutions are evaluated. The personal-best position of each agent and the global-best solution of the population are then updated according to the newly obtained fitness values. The iterative process continues until the maximum iteration number is reached, at which point the best candidate solution * xbest* is returned. This hybrid structure yields a more robust optimization process in comparison with the original ALA, particularly in scenarios involving nonlinear, multimodal, or stiff search spaces. The synergy between ALA’s behavioral diversity and the gbest-guided memory mechanism provides enhanced convergence speed, improved exploitation capability, and better solution reliability, as later demonstrated by the simulation results.

## **Continuous stirred tank reactor (CSTR) system**

**Fig. 2:**
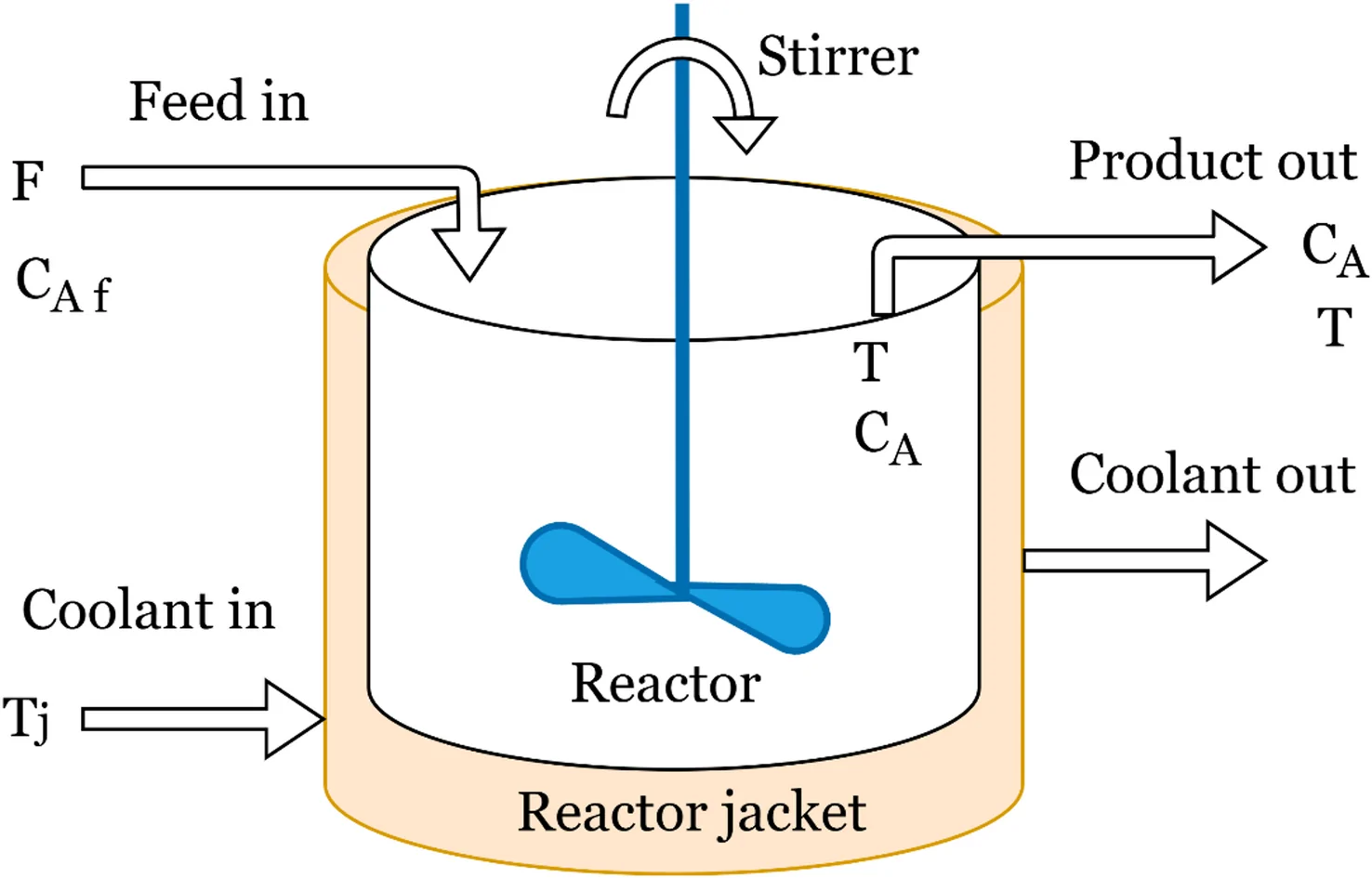
Schematic representation of the jacketed CSTR system.

The nonlinear process investigated in this study is a jacketed continuous stirred tank reactor (CSTR), depicted schematically in Fig. 2. The reactor operates with a constant liquid volume, as the inlet and outlet flow rates are equal, and mechanical agitation ensures perfect mixing of the reacting fluid. Under this assumption, the temperature * T* and concentration $\:{C}_{A}$ inside the reactor are spatially uniform and identical to those in the outlet stream. The system performs an irreversible, first-order exothermic reaction $\:A\to\:B$, and the heat released by the reaction is removed through an external cooling jacket. The jacket temperature $\:{T}_{j}$ acts as the manipulated input and is used to regulate the reactor temperature * T*.

### Modeling assumptions and reaction kinetics

Following the classical diabatic CSTR formulation of Bequette^2^, the model is constructed under standard assumptions: perfect liquid-phase mixing, constant reactor volume, constant physical properties, negligible vapor-phase effects, and direct manipulation of the jacket temperature. The reaction rate per unit volume follows the Arrhenius law:

11 $$\:{R}_{A}={k}_{0}exp\left(-\frac{E}{RT}\right){C}_{A}$$

where $\:{R}_{A}$ denotes the reaction-rate term, $\:{k}_{0}$ is the pre-exponential factor and * E* the activation energy. This exponential temperature dependence introduces pronounced nonlinearity, making the reactor highly sensitive to thermal disturbances.

### Dynamic model: material and energy balances

The transient behavior of the concentration and temperature states is obtained by applying mass and energy conservation principles. The material balance for species * A* yields

12 $$\:\frac{{dC}_{A}}{dt}=\frac{F}{V}\left({C}_{Af}-{C}_{A}\right)-{k}_{0}{e}^{-E/\left(RT\right)}{C}_{A}$$

while the reactor temperature satisfies the energy balance

13 $$\:\rho\:{c}_{p}\frac{dT}{dt}=\frac{F\rho\:{c}_{p}}{V}\left({T}_{f}-T\right)+\left(-\varDelta\:H\right){k}_{0}{e}^{-E/\left(RT\right)}{C}_{A}-\frac{UA}{V}\left(T-{T}_{j}\right)$$

The three terms on the right-hand side of Eq. (13) correspond to heat entering with the feed stream, heat generated by the exothermic reaction, and heat removed through the cooling jacket, respectively. Equations (12)-(13) represent the standard nonlinear CSTR model widely used in dynamic analysis and control design.

### Model parameters and steady-state operating point

The numerical parameters of the CSTR correspond to the benchmark configuration reported by Henson and Seborg^3^. The reactor operates with a volumetric feed flow rate of * F=100* L/min and a liquid reactor volume of * V=100* L. The feed stream enters with a reactant concentration of $\:{C}_{Af}=1$ mol/L at a temperature of $\:{T}_{f}=350$ K. The intrinsic kinetics follow an Arrhenius form with a pre-exponential factor of $\:{k}_{0}=7.2\times\:{10}^{10}$ min^−1^ and * E/R=8750* K. The thermophysical properties of the reacting mixture are assumed constant, with a density of $\:\rho\:=1000$ g/L, a specific heat capacity of $\:{c}_{p}=0.239$ J/g∙K, and a reaction enthalpy of $\:-\varDelta\:H=50000$ J/mol. Heat is exchanged with the surrounding cooling jacket through an overall heat-transfer coefficient-area product of * UA=50000* J/min·K.

The nominal steady-state operating point is obtained by solving Eqs. (12)-(13) under the equilibrium conditions $\:{dC}_{A}/dt=0$ and * dT/dt=0*. For the above parameter set, the steady-state solution is $\:{T}_{j}=280$ K, * T=304.167553089807* K and $\:{C}_{A}=0.977403565332$ mol/L. This operating point corresponds to a thermally stable regime of the reactor and serves as the reference around which the controller is optimized and evaluated.

## Fundamental of PID with filter (PIDf) controller

The PID controller structure is employed as the baseline feedback controller due to its well-established industrial relevance and compatibility with optimization-based tuning frameworks in this study^20^. In its classical form, the controller generates the manipulated signal based on a weighted combination of the instantaneous control error, its accumulated integral, and its rate of change. This structure allows the proportional term to provide immediate corrective action, the integral term to eliminate steady-state errors, and the derivative term to enhance the transient response by predicting future error trends.

Although the derivative action improves closed-loop stability and responsiveness, its direct implementation is highly sensitive to high-frequency measurement noise. This sensitivity is particularly critical in thermally driven nonlinear processes such as CSTRs, where temperature measurements may contain fluctuations that lead to excessive control effort when an unfiltered derivative term is used. To alleviate this issue, practical PID realizations incorporate a first-order low-pass filter in the derivative channel, resulting in the so-called PID with filter (PIDf) formulation. The transfer function of the PIDf controller used in this study is expressed as:

14 $$\:C\left(s\right)=\frac{U\left(s\right)}{E\left(s\right)}={K}_{p}+\frac{{K}_{i}}{s}+{K}_{d}\frac{Ns}{s+N}$$

where $\:{K}_{p}$, $\:{K}_{i}$ and $\:{K}_{d}$ denote the proportional, integral, and derivative gains, respectively, and * N* represents the derivative-filter coefficient. The term * Ns/(s+N)* acts as a filtered approximation of the ideal derivative operator, reducing noise amplification while preserving sufficient phase lead for improved transient behavior. The filter coefficient * N* determines the bandwidth of the derivative action and is typically selected several times larger than the closed-loop bandwidth to achieve an appropriate balance between noise attenuation and responsiveness.

This filtered PID structure is particularly suitable for nonlinear CSTR temperature regulation, where abrupt changes in heat-release rates may occur and the measurement noise can adversely influence the derivative term. By mitigating these high-frequency components, the PIDf controller provides a smoother and more robust manipulated-input signal while retaining the desirable predictive nature of derivative action. Consequently, the set of tunable parameters ($\:{K}_{p}$, $\:{K}_{i}$, $\:{K}_{d}$, * N*) forms a four-dimensional search space for optimization, enabling advanced meta-heuristic algorithms to design a controller that enhances both stability and transient performance of the nonlinear reactor system.

## Simulation results

This section presents a comprehensive evaluation of the proposed gbest-ALA-tuned PIDf controller through numerical simulations performed on the nonlinear CSTR model. The controller performance is assessed under reference-tracking scenarios and compared against several state-of-the-art metaheuristic tuning methods as well as classical PIDf tuning approaches. The simulation study is structured to ensure a fair and systematic comparison by employing identical operating conditions, parameter bounds, population sizes, and iteration numbers for all optimization-based methods.

The analysis begins with the definition of the objective function and the implementation details of the proposed gbest-ALA framework. Subsequently, comparative tuning strategies are introduced, followed by statistical verification based on multiple independent runs. The convergence characteristics of the optimization processes, the resulting optimal PIDf parameters, and the closed-loop transient responses are then examined in detail. Finally, setpoint-tracking performance is evaluated using standard error-based performance indices, alongside the dynamic behavior of the jacket temperature and reactant concentration. A detailed discussion of the obtained results is deferred to Sect. 6.7.

### Objective function definition and application of gbest-ALA

To tune the PIDf controller parameters using the proposed gbest-ALA optimizer, a scalar performance index is defined to quantify the closed-loop temperature regulation quality of the nonlinear CSTR. In this study, the integral of squared error (ISE) is adopted as the objective function, which is one of the most widely used criteria in control-system design due to its strong penalization of large transient deviations^41^. The ISE criterion was preferred because the squared-error term assigns greater weight to large instantaneous tracking errors than absolute-error-based criteria. This property is particularly relevant for nonlinear exothermic CSTR temperature regulation, where excessive transient temperature deviations may adversely affect product quality and safe process operation. Therefore, ISE provides a suitable single-objective tuning criterion for the step-response-based optimization considered in this study. It should also be noted that IAE, ITAE, and ITSE are not used as optimization objectives; instead, they are employed later as complementary evaluation metrics to assess the closed-loop tracking performance from different perspectives:

15 $$\:ISE=\underset{0}{\overset{{t}_{f}}{\int\:}}{e}^{2}\left(t\right)dt$$

where $\:e\left(t\right)={T}_{ref}\left(t\right)-T\left(t\right)$ denotes the reactor temperature tracking error and the evaluation horizon is selected as $\:{t}_{f}=20$ min. During the objective-function evaluation, the reactor temperature reference is subjected to a step increase of 10 K: specifically, the reference is raised from the nominal steady-state value * T=304.16755* K to $\:{T}_{ref}=314.16755$ K at * t=1* min. This setpoint change is deliberately introduced to sufficiently excite the nonlinear dynamics of the CSTR and to evaluate the transient response, tracking accuracy, and regulation capability of each candidate PIDf parameter set.

The PIDf controller parameters optimized by the proposed gbest-ALA are $\:\left\{{K}_{p},{K}_{i},{K}_{d},\:N\right\}$. The corresponding lower and upper bounds defining the feasible search space of the optimizer are summarized in Table 1. These bounds are selected to ensure physically meaningful controller gains while allowing sufficient flexibility for the metaheuristic algorithm to explore and exploit the parameter space effectively. In practical engineering applications, the search ranges of PIDf parameters can be determined based on prior process knowledge, actuator limitations, desired closed-loop response characteristics, and preliminary tuning results obtained from classical tuning rules or model-based PID tuning tools. The lower and upper bounds should be selected to exclude impractical controller settings while still providing a sufficiently wide search region for the optimizer. In particular, very small gain values may result in weak control action and slow tracking performance, whereas excessively large gain values may cause aggressive control effort, overshoot, oscillatory behavior, actuator saturation, or numerical instability. For the derivative-filter coefficient (* N*), the range should be selected to maintain an appropriate balance between derivative action and noise attenuation, since a very large * N* may increase sensitivity to high-frequency measurement noise, while a very small * N* may excessively weaken the derivative contribution. Therefore, the parameter bounds should be defined as a compromise between practical feasibility, closed-loop stability, control-effort limitations, and sufficient search-space flexibility. All optimization-based tuning methods considered in this study employ the same parameter limits to guarantee a fair comparison.

**Table 1:** Lower and upper bounds of PIDf controller parameters used in the optimization process.

Parameter	Lower	Upper
$\:{K}_{p}$	0.05	1
$\:{K}_{i}$	0.1	2
$\:{K}_{d}$	0.02	0.4
* N*	1	10

**Fig. 3:**
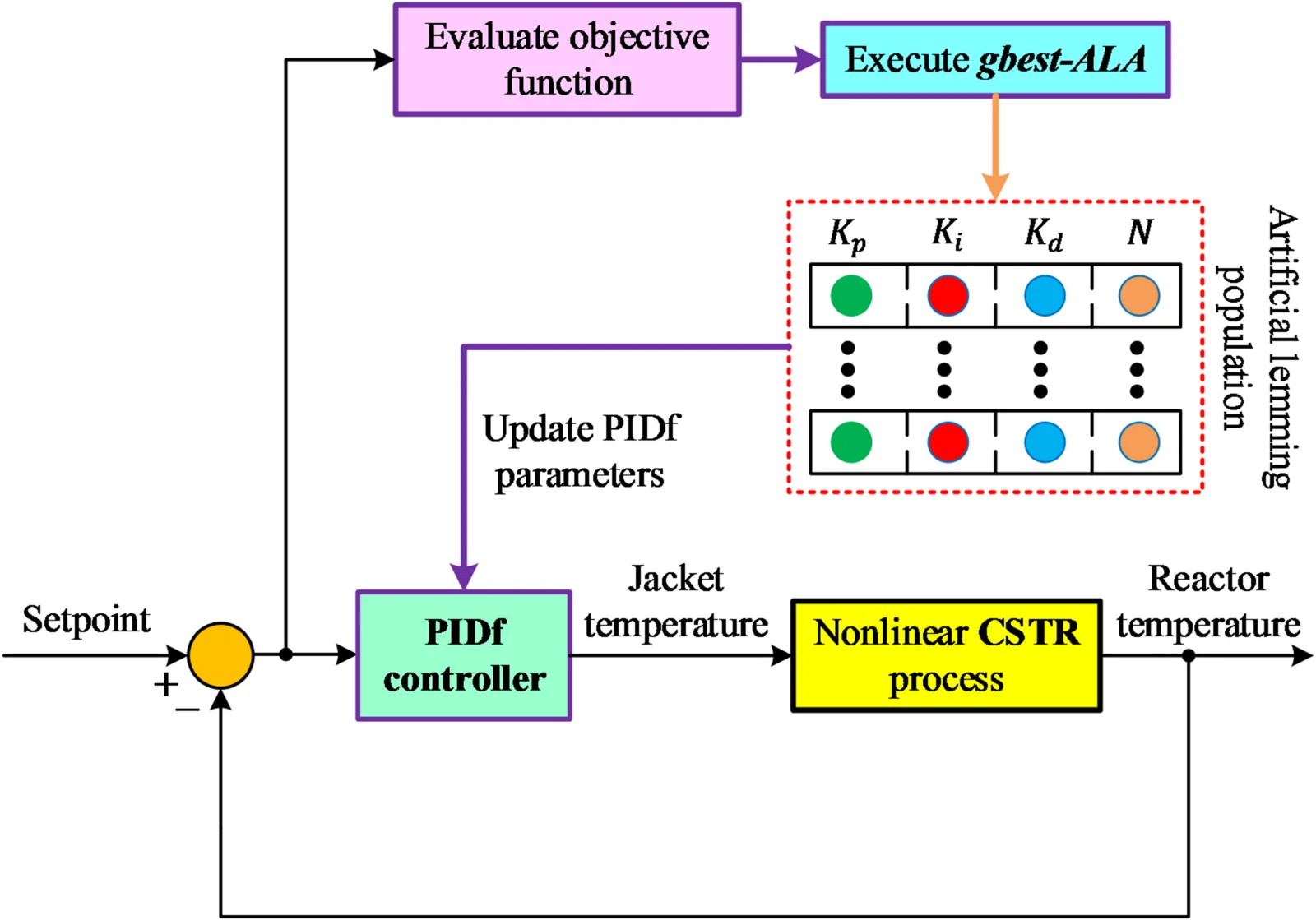
Block diagram of the gbest-ALA-based PIDf tuning framework for nonlinear CSTR temperature control.

Following the definition of the objective function and the parameter bounds, the overall optimization and control framework is illustrated in Fig. 3. For each candidate solution generated by gbest-ALA, the nonlinear CSTR model is simulated in closed loop with the corresponding PIDf parameters under the specified reference change. The resulting ISE value is then evaluated and fed back to the optimizer, which iteratively updates the population until the termination criterion is satisfied. The PIDf parameter set yielding the minimum ISE is finally selected as the optimal controller for subsequent performance comparisons.

### Compared tuning methods

To assess the effectiveness of the proposed gbest-ALA-based PIDf tuning strategy, its performance is compared with a set of recently developed metaheuristic algorithms as well as widely used classical tuning approaches. The selected metaheuristic methods include the original ALA^33^, TOC^34^, MSO^35^ and PGA^36^. These algorithms were all introduced in 2025 and published in high-impact journals, making them representative of the current state of the art in metaheuristic-based controller tuning.

For a fair and unbiased comparison, all metaheuristic algorithms are executed under identical computational settings. Specifically, the population size is fixed at * M=20*, the maximum number of iterations is set to $\:{T}_{max}=50$, and each algorithm is independently run 25 times to account for stochastic variations. The same objective function, parameter bounds, stopping criteria, and simulation conditions are applied across all methods. This unified configuration ensures that any observed performance differences arise solely from the intrinsic search mechanisms of the algorithms rather than from unequal parameterization.

In addition to metaheuristic-based tuning strategies, two classical PIDf tuning approaches are included as baseline references. The first is the built-in Simulink PIDf Tuner^42^, which provides an automated model-based tuning solution commonly used in industrial control applications. The second is the well-established Tyreus-Luyben method^43^, a classical analytical tuning rule commonly adopted as a more conservative and robust alternative to conventional Ziegler-Nichols-type approaches. The inclusion of these classical methods enables a clear comparison between modern optimization-driven designs and traditional tuning techniques.

The results obtained from all tuning methods are evaluated using identical performance metrics and are presented in the following subsections, where convergence behavior, optimal parameter values, and closed-loop dynamic responses are systematically analyzed.

### Statistical verification of proposed gbest-ALA

**Fig. 4:**
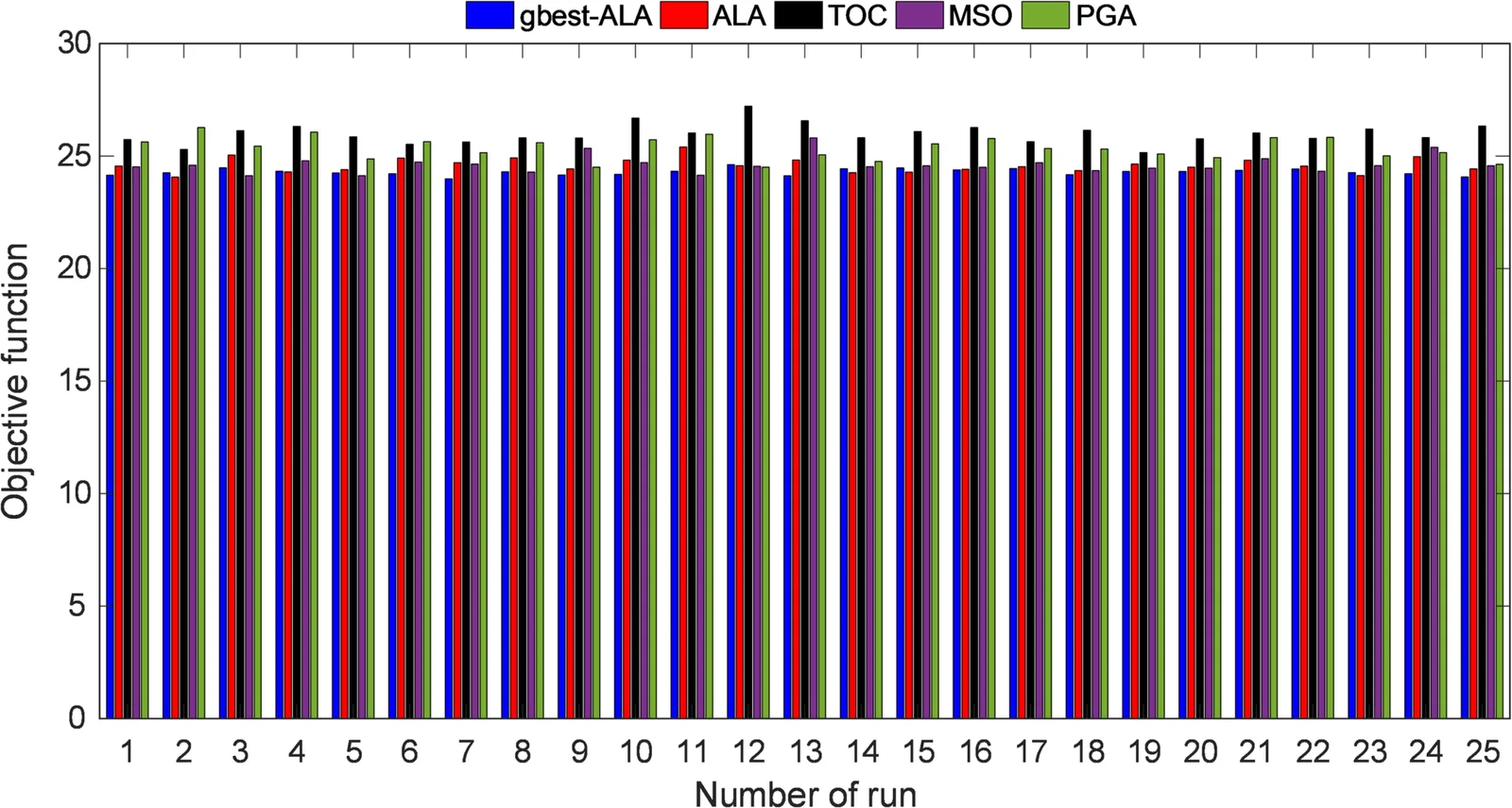
Objective-function values obtained over 25 independent runs for all compared metaheuristic algorithms.

To statistically verify the effectiveness and reliability of the proposed gbest-ALA-based tuning strategy, all metaheuristic algorithms are executed over 25 independent runs under identical conditions. The resulting objective-function values obtained in each run are illustrated in Fig. 4, while a comprehensive statistical summary is reported in Table 2.

As depicted in Fig. 4, the gbest-ALA (blue bars) consistently yields the lowest objective-function values across the majority of independent runs. Although a small number of runs exhibit marginally lower values for the original ALA (red bars), the overall trend clearly indicates the superior robustness and stability of gbest-ALA. In contrast, TOC (black bars) frequently produces the highest objective-function values, particularly toward the later runs, highlighting its comparatively weaker performance under the same optimization settings. By the end of the 25 runs, gbest-ALA achieves the lowest final objective value among all considered algorithms, whereas TOC exhibits the highest.

The quantitative statistical measures presented in Table 2 further substantiate these observations. The proposed gbest-ALA attains the lowest best, mean, and median objective-function values, indicating superior optimization accuracy and consistency. Moreover, it exhibits the smallest standard deviation, which reflects reduced performance variability and enhanced robustness across repeated runs. The original ALA ranks second, while MSO and PGA demonstrate intermediate performance levels. TOC yields the largest dispersion and the poorest average results, resulting in the lowest overall rank.

**Table 2:** Statistical comparison of objective-function values obtained from 25 independent runs.

Measure	gbest-ALA	ALA	TOC	MSO	PGA
Best	**23.9869**	24.0653	25.1572	24.1316	24.5075
Worst	**24.6202**	25.4033	27.2176	25.8063	26.2696
Mean	**24.2916**	24.5953	25.9868	24.6307	25.3469
Median	**24.3035**	24.5538	25.8566	24.5708	25.3408
SD	**0.1449**	0.3131	0.4417	0.3930	0.4922
Elapsed time (s)	677.0196	**634.0792**	895.6704	706.1588	788.7523
Rank	**1**	2	5	3	4

Based on these statistical indicators, the proposed gbest-ALA achieves the top overall ranking among all compared metaheuristic algorithms. This outcome confirms that the integration of the gbest operator significantly enhances the search stability and convergence reliability of the original ALA framework under identical computational conditions. In addition to the objective-function statistics, the average elapsed time per independent run was recorded over 25 trials and added to Table 2. The proposed gbest-ALA requires an average elapsed time of 677.0196 s, which is slightly higher than that of the original ALA due to the additional global-best refinement step. However, this increase remains limited, and gbest-ALA requires less average computational time than TOC, MSO, and PGA under the same simulation conditions. Therefore, the improved statistical performance and convergence reliability of gbest-ALA are achieved with an acceptable computational burden.

### Evolution of objective function and obtained best PIDf parameters

**Fig. 5:**
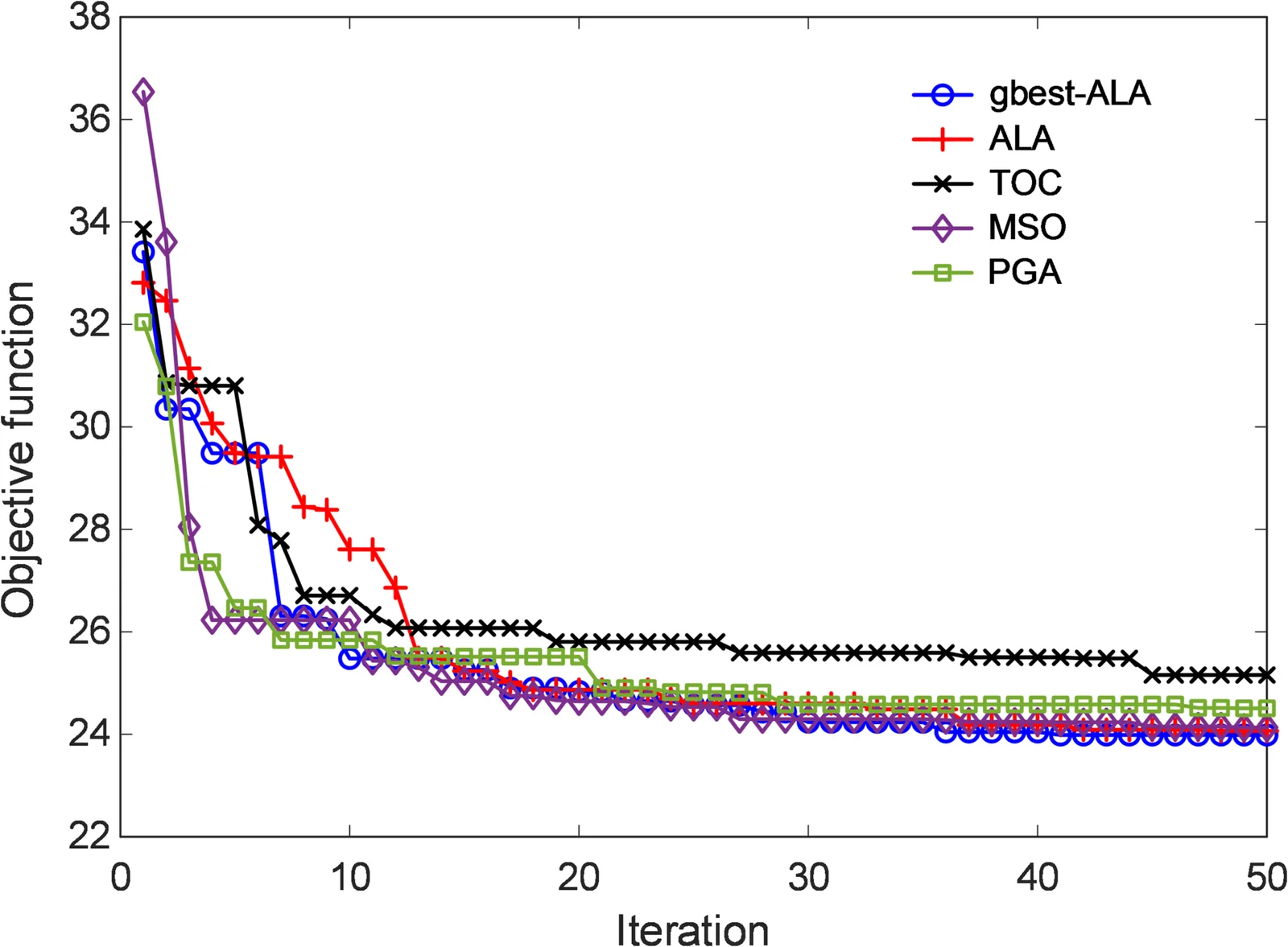
Convergence curves of the objective-function values for the compared metaheuristic algorithms.

The convergence characteristics of the proposed gbest-ALA and the compared metaheuristic algorithms are illustrated in Fig. 5, where the evolution of the objective-function value over the optimization iterations is depicted. All algorithms are executed under identical conditions with a population size of * M=20* and a maximum iteration number of $\:{T}_{max}=50$, ensuring a fair assessment of convergence speed and stability.

As observed in Fig. 5, the proposed gbest-ALA exhibits a rapid reduction of the objective-function value during the early iterations, followed by a smooth and stable convergence toward the lowest final value. The original ALA demonstrates a comparable initial descent but converges to a slightly higher objective value. In contrast, TOC shows a slower convergence rate and settles at a higher plateau, while MSO and PGA display intermediate convergence behavior. The monotonic and stable convergence profile of gbest-ALA indicates an improved balance between exploration and exploitation, resulting in enhanced convergence reliability under identical computational settings.

The optimal PIDf controller parameters obtained using each tuning method are summarized in Table 3. These parameter sets correspond to the best-performing solutions identified at the end of the optimization process or, for classical approaches, to the final tuned values produced by the respective tuning rules.

**Table 3:** Obtained optimal PIDf parameters.

Tuning method	$\:{K}_{p}$	$\:{K}_{i}$	$\:{K}_{d}$	* N*
gbest-ALA	0.9976	1.9983	0.2280	9.7094
ALA	0.9567	1.8734	0.3969	9.1043
TOC	0.9218	1.8015	0.3953	9.6950
MSO	0.9107	1.9394	0.3768	8.5781
PGA	0.9763	1.8969	0.2907	9.0339
Simulink PIDf tuner	0.4603	1.1690	0.3523	6.4869
Tyreus-Luyben	0.1203	1.4657	0.1058	10

As reported in Table 3, the gbest-ALA-tuned controller yields parameter values that differ noticeably from those obtained using both the original ALA and the other metaheuristic algorithms, as well as from classical tuning approaches. The Simulink PIDf tuner and the Tyreus-Luyben method provide considerably different gain magnitudes, reflecting their fundamentally distinct tuning philosophies compared to optimization-based strategies. These optimal parameter sets are subsequently employed in the closed-loop simulations to evaluate transient performance and tracking accuracy in the following subsection.

### Closed-loop response

**Fig. 6:**
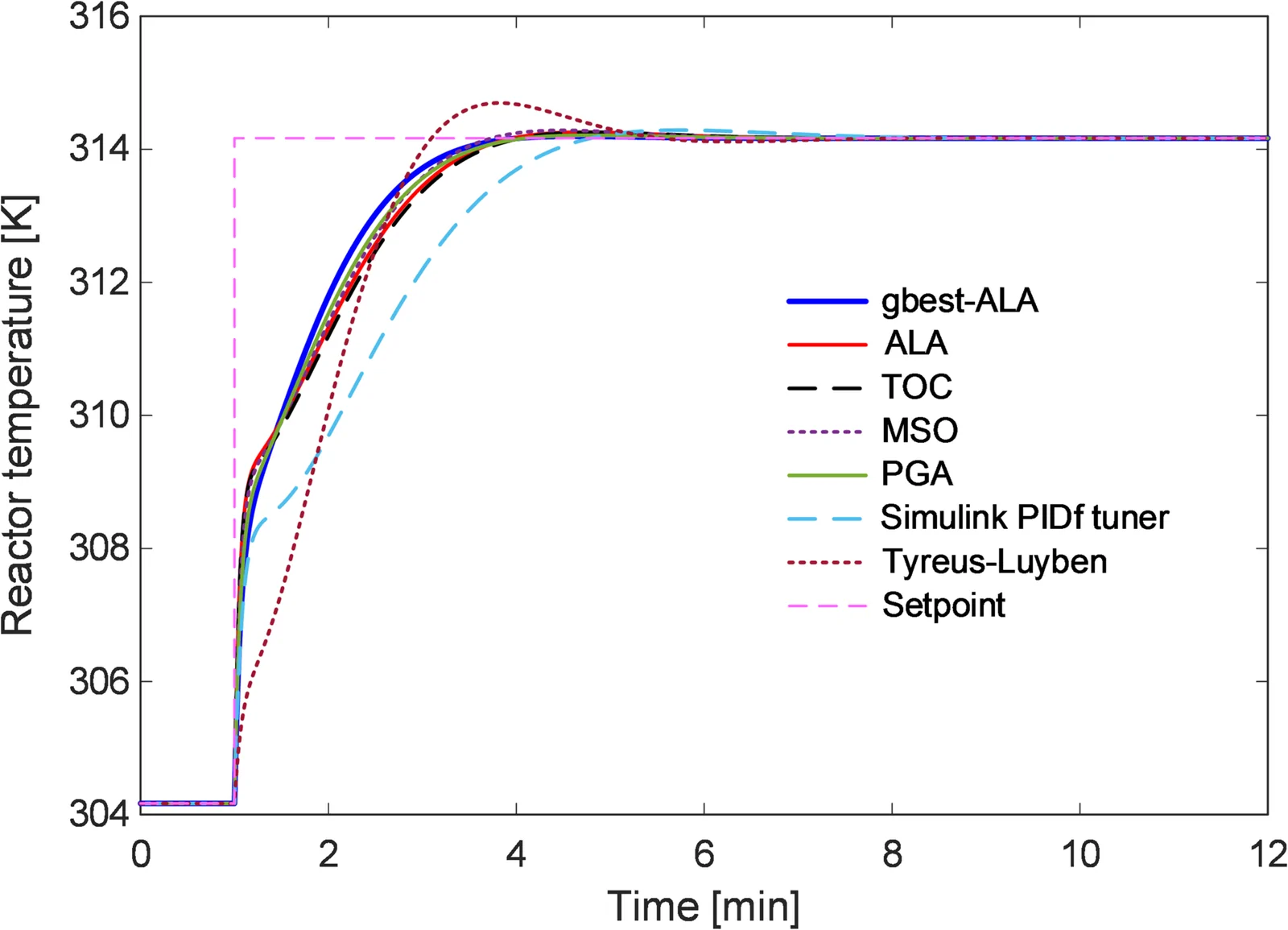
Closed-loop reactor temperature responses to a 10 K step change.

The closed-loop temperature regulation performance of the nonlinear CSTR is evaluated under a step change in the reactor temperature reference. The applied setpoint is defined as: $\:{T}_{ref}\left(t\right)=304.16755+10u(t-1)$ K, corresponding to a 10 K increase at * t=1* min. This excitation is selected to assess the transient tracking capability, overshoot characteristics, and settling behavior of the PIDf controllers tuned by the compared methods. The resulting reactor temperature responses are illustrated in Fig. 6, where the full time-domain behavior of all controllers is shown. The comparison includes both metaheuristic-based tuning methods and classical PIDf tuning approaches.

**Fig. 7:**
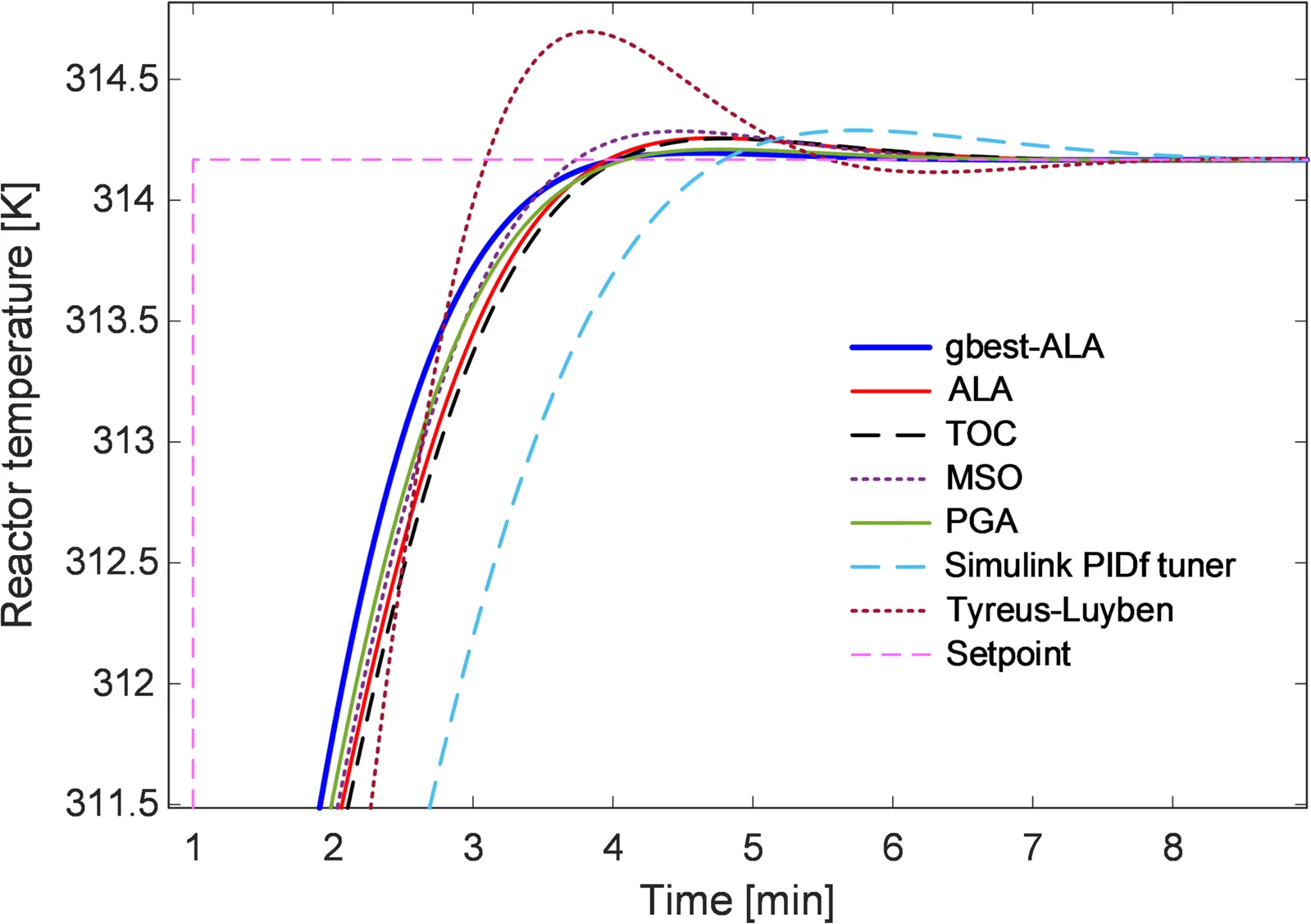
Zoomed view of the closed-loop reactor temperature responses.

As observed in Fig. 6, all tuning strategies are able to regulate the reactor temperature toward the new setpoint; however, noticeable differences arise in terms of rise time, overshoot, and settling time characteristics. To provide a clearer comparison of the transient region, a magnified view of the response around the setpoint transition is presented in Fig. 7.

The quantitative transient-response indices extracted from these simulations are summarized in Table 4, including rise time, settling time, peak temperature, and overshoot. The settling time is computed using a ± 0.2 K tolerance band around the final setpoint value, ensuring a consistent and practically relevant performance assessment for all controllers.

**Table 4:** Transient performance indices of the closed-loop reactor temperature responses.

Tuning method	Rise time (min)	Settling time (min)	Peak (K)	Overshoot (K)
gbest-ALA	1.5631	3.3403	314.1954	0.0278
ALA	1.8004	3.5317	314.2571	0.0895
TOC	1.8565	3.6008	314.2551	0.0876
MSO	1.7213	3.3814	314.2852	0.1176
PGA	1.6971	3.4885	314.2101	0.0425
Simulink PIDf tuner	2.5321	4.3566	314.2889	0.1214
Tyreus-Luyben	1.6301	4.8279	314.6971	0.5296

As reported in Table 4, the PIDf controller tuned by the proposed gbest-ALA achieves the shortest rise time and the smallest overshoot among all considered methods, while maintaining a rapid settling behavior within the specified tolerance band. The original ALA, MSO, and PGA exhibit comparable responses with slightly larger overshoot values, whereas TOC and the Simulink PIDf tuner display slower settling dynamics. The Tyreus-Luyben-tuned controller shows the largest overshoot and the longest settling time, reflecting its more conservative tuning characteristics. A detailed interpretation of these results is provided in Sect. 6.7.

### Setpoint tracking

**Fig. 8:**
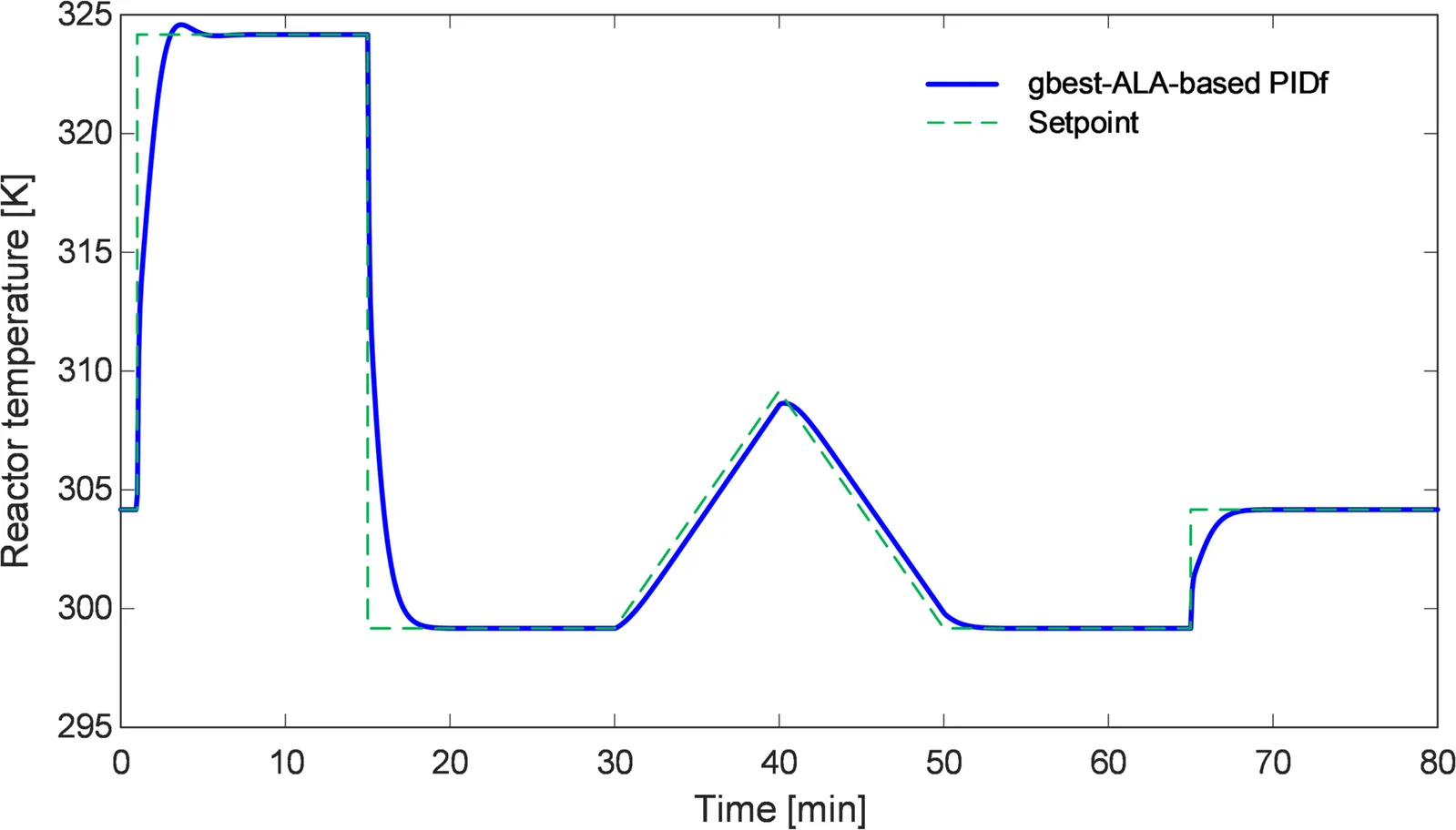
Setpoint-tracking response of the reactor temperature obtained using the gbest-ALA-based PIDf controller.

Beyond single-step regulation, the tracking capability of the tuned controller is further examined under a multi-segment setpoint profile over a longer horizon. In this test, the reactor temperature reference is changed sequentially, and the resulting closed-loop tracking response of the gbest-ALA-based PIDf controller is depicted in Fig. 8.

As observed in Fig. 8, the proposed controller follows the scheduled setpoint transitions with stable behavior and without sustained oscillations, indicating adequate tracking performance over the entire $\:{t}_{f}=80$ min evaluation window.

#### Comparison of error-based performance indices

To quantitatively evaluate tracking quality under the setpoint profile in Fig. 8, standard error-based performance indices are computed using the general form:

16 $$\:{P}_{index}=\underset{0}{\overset{{t}_{f}}{\int\:}}{t}^{m}{\left|e\left(t\right)\right|}^{n}dt$$

where $\:e\left(t\right)={T}_{ref}\left(t\right)-T\left(t\right)$ and $\:{t}_{f}=80$ min. By selecting * (m,n)*, four widely used indices are obtained: the integral of absolute error (*IAE*) with * (m=0,n=1)*, the integral of squared error (* ISE*) with * (m=0,n=2)*, the integral of time-weighted absolute error (* ITAE*) with * (m=1,n=1)*, and the integral of time-weighted squared error (* ITSE*) with * (m=1,n=2)*. These indices are evaluated numerically for each tuning method based on the same setpoint-tracking scenario. The computed values of * IAE*, * ISE*, * ITAE*, and * ITSE* are summarized in Table 5.

**Table 5:** Comparison of error-based performance indices.

Tuning method	* IAE*	* ISE*	* ITAE*	* ITSE*
gbest-ALA	**39.4642**	228.6113	**933.9974**	**2.7652E + 03**
ALA	42.9602	**227.5701**	1.0084E + 03	2.8066E + 03
TOC	44.6118	237.9552	1.0480E + 03	2.9458E + 03
MSO	42.0128	228.1805	979.1505	2.7880E + 03
PGA	41.7041	232.5481	986.2454	2.8427E + 03
Simulink PIDf tuner	69.0262	428.8558	1.6231E + 03	5.4667E + 03
Tyreus-Luyben	63.1614	512.4381	1.3623E + 03	6.0067E + 03

As reported in Table 5, the proposed gbest-ALA achieves the lowest values for * IAE*, * ITAE* and * ITSE*, indicating improved overall tracking accuracy and reduced time-weighted deviations over the full evaluation horizon. Notably, the lowest * ISE* value is attained by the original ALA, while gbest-ALA remains a close competitor in terms of squared-error accumulation. This outcome should be interpreted in view of the difference between the tuning and validation scenarios. The PIDf parameters were optimized under the single-step 20 min scenario defined in Sect. 6.1, whereas Table 5 corresponds to a different 80 min multi-segment setpoint-tracking validation scenario. Therefore, the controller parameters obtained from the single-step * ISE* minimization are not necessarily expected to be globally optimal with respect to * ISE* under a different reference trajectory and evaluation horizon. In addition, the * ISE* difference between ALA and gbest-ALA in Table 5 is relatively small, approximately 0.46%, while gbest-ALA provides lower * IAE*, * ITAE*, and * ITSE* values. This observation highlights that different indices may emphasize different aspects of tracking performance; therefore, multiple criteria are considered for a balanced assessment.

#### Changes of jacket temperature $\:{\boldsymbol{T}}_{\boldsymbol{j}}$ and concentration of $\:\boldsymbol{A}$ in reactor $\:{\boldsymbol{C}}_{\boldsymbol{A}}$

**Fig. 9:**
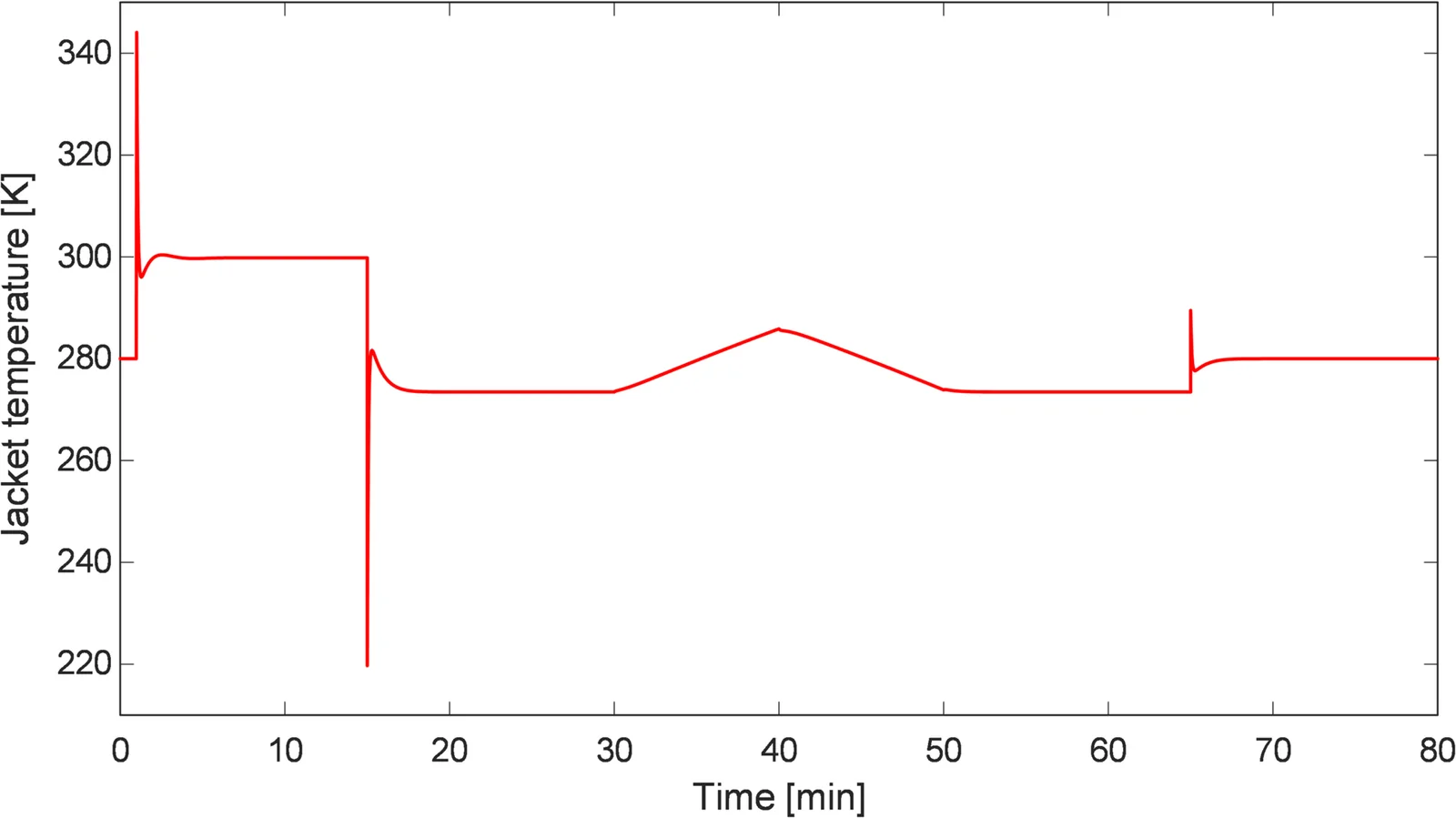
Closed-loop response of the jacket temperature $\:{T}_{j}$.

**Fig. 10:**
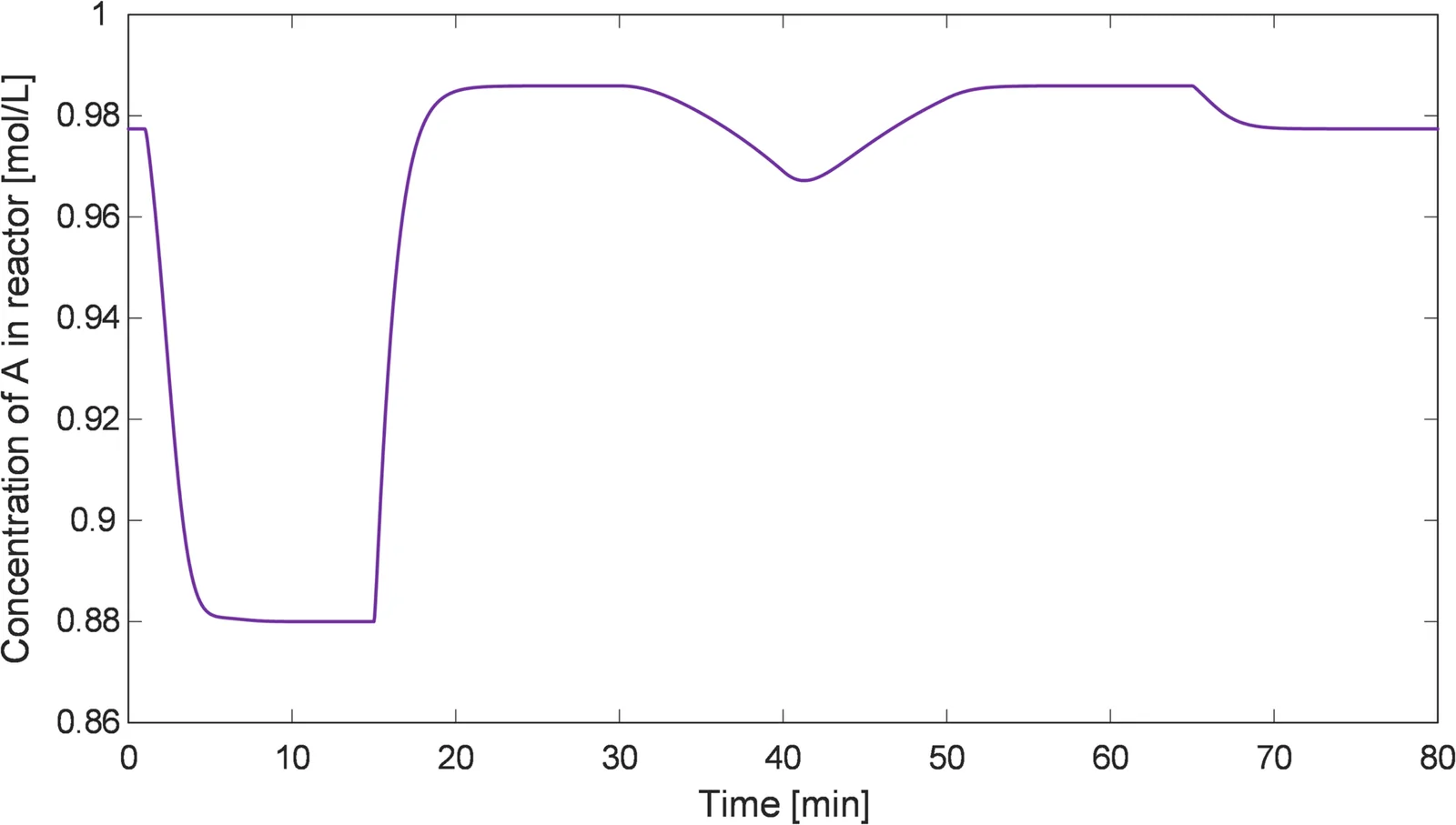
Closed-loop response of the reactant concentration $\:{C}_{A}$.

In addition to the reactor temperature tracking error, the closed-loop variations of the manipulated variable and the concentration state are investigated under the same setpoint profile of Fig. 8. Specifically, the time evolution of the jacket temperature $\:{T}_{j}$ (controller output) is presented in Fig. 9, and the corresponding concentration trajectory of reactant * A* in the reactor, $\:{C}_{A}$, is shown in Fig. 10.

As seen in Fig. 9, $\:{T}_{j}$ adjusts dynamically to compensate for the scheduled reference changes, providing the required heat-removal or heat-supply action to maintain the reactor temperature close to the desired setpoint. In parallel, Fig. 10 demonstrates that the reactant concentration varies without sustained oscillations during the temperature transitions and remains within a physically consistent range throughout the tracking experiment, reflecting stable closed-loop operation of the nonlinear CSTR under the proposed control strategy.

### Discussion

The simulation results collectively indicate that the proposed gbest-ALA-based PIDf controller provides a consistent and effective solution for temperature regulation of the nonlinear CSTR. Across different evaluation perspectives (including statistical robustness, convergence behavior, transient performance, and long-horizon setpoint tracking), the gbest-ALA exhibits competitive performance compared to both recently developed metaheuristic-based tuning methods and classical PIDf tuning approaches. From an optimization standpoint, the statistical verification and convergence analyses suggest that incorporating the gbest operator into the original ALA improves search stability and convergence reliability. The reduced variability across independent runs and the smoother convergence profile indicate that the proposed modification enhances the balance between exploration and exploitation without increasing computational complexity. These characteristics are particularly beneficial for controller tuning problems involving nonlinear dynamics and multimodal objective landscapes, such as those encountered in CSTR temperature control.

In the time-domain analysis, the gbest-ALA-tuned PIDf achieves a fast response with low overshoot and short settling time, while maintaining stable closed-loop behavior. Although certain performance indices (notably the * ISE*) are slightly lower for the original ALA in the setpoint-tracking test, the gbest-ALA attains improved values for several other error-based indices, including * IAE*, * ITAE*, and * ITSE*. This behavior can be explained by the fact that the PIDf parameters were optimized for a single-step * ISE*-based tuning scenario, whereas the multi-segment setpoint-tracking test was used as a separate validation scenario with a different reference trajectory and evaluation horizon. This observation highlights the importance of employing multiple complementary performance measures; as different indices emphasize different aspects of control performance.

The setpoint-tracking experiments further indicate that the proposed controller maintains stable operation under sequential reference changes. The corresponding jacket-temperature and concentration responses remain bounded and physically consistent, suggesting that improved temperature regulation is achieved without inducing excessive control action or undesirable effects on reactor concentration dynamics. This behavior is particularly relevant for practical CSTR operation, where large control signals or significant concentration deviations may be undesirable.

Overall, the discussion suggests that the proposed gbest-ALA framework provides a balanced and reliable tuning strategy for PIDf controllers in nonlinear chemical processes. Its advantages are reflected in convergence consistency and overall closed-loop performance, making it a competitive alternative to both conventional tuning rules and recently proposed metaheuristic methods for CSTR temperature control. It is also important to clarify the position of the proposed PIDf framework relative to predictive control approaches such as MPC and enhanced predictive functional control. Predictive control methods are highly effective for complex nonlinear processes, particularly when explicit constraint handling, multivariable interactions, prediction horizons, and time-delay compensation are central design requirements. However, these advantages may require an accurate prediction model, additional design parameters, online predictive calculations, and higher implementation effort. In contrast, the proposed optimization-assisted PIDf framework preserves the structural simplicity, transparency, and industrial familiarity of PID-based control while improving the tuning quality through the gbest-ALA optimizer. Therefore, the proposed method should not be interpreted as a replacement for MPC/PFC in all nonlinear process-control applications, but rather as a practical PID-compatible alternative for cases where ease of implementation, controller transparency, and low online computational burden are prioritized. It should be noted that the evaluation is conducted under setpoint variations and nominal operating conditions. Further investigation under explicit disturbance scenarios and parametric uncertainties may provide additional insights into the robustness characteristics of the proposed approach.

## Conclusion

This study presented an optimization-assisted PIDf tuning framework for nonlinear CSTR temperature regulation based on a global-best guided artificial lemming algorithm. By incorporating a global-best guidance mechanism into the original search process, the proposed approach enhances convergence stability and solution consistency while preserving population diversity, which is important for handling nonlinear control landscapes. The effectiveness of the proposed PIDf tuning strategy was evaluated on a nonlinear jacketed CSTR model under setpoint variations and nonlinear operating conditions. Comparative analyses with recently developed metaheuristic optimization methods and classical PID tuning approaches indicate that the proposed framework achieves competitive convergence behavior and improved closed-loop temperature regulation performance.

Overall, the proposed framework maintains the structural simplicity and transparency of PID-based controllers while benefiting from optimization-assisted parameter tuning. The results suggest that the approach provides a consistent and practical solution for temperature regulation in nonlinear CSTR systems. Future work may focus on extending the proposed framework toward experimental validation, real-time implementation, and further analysis under disturbance conditions and parametric uncertainties.

## Data Availability

The datasets generated and analyzed during the current study are available from the corresponding author on reasonable request.
